# Phytoglobin 1 in plant hypoxia acclimation and development: integrating oxygen sensing, nitric oxide homeostasis, and redox balance

**DOI:** 10.1093/jxb/erag147

**Published:** 2026-03-19

**Authors:** Elmar van der Wijk, Jian Xu, Rashmi Sasidharan

**Affiliations:** Department of Plant and Animal Biology, Radboud Institute for Biological and Environmental Sciences, Radboud University, AJ Nijmegen 6525, The Netherlands; Department of Plant and Animal Biology, Radboud Institute for Biological and Environmental Sciences, Radboud University, AJ Nijmegen 6525, The Netherlands; Plant Stress Resilience, Institute of Environmental Biology, Utrecht University, CH Utrecht 3584, The Netherlands; Nagoya University, Japan

**Keywords:** ERF-VII, ethylene, flooding, hypoxia, mitochondrial retrograde response, nitric oxide, oxidative stress, PGB1, phytoglobin, ROS

## Abstract

Hypoxia is integral to the plant life cycle, occurring during both development and environmental stresses such as flooding. The class I phytoglobins (PGB1s) have emerged as important regulators of plant hypoxia responses in both these contexts due to their multifaceted roles in nitric oxide (NO) and reactive oxygen species (ROS) homeostasis, and alternative energy generation. Physiological *PGB1* expression overlaps with developmental hypoxic niches, facilitating hypoxic energy generation through the PGB1–NO cycle. Sustained induction of PGB1 by various signals during the progression of a flooding event reflects its important but potentially distinct roles in flooding stress acclimation. These include short-term PGB1-mediated NO scavenging to stabilize ETHYLENE RESPONSE FACTOR VII transcription factors, and in the long-term hypoxic energy generation, oxidative stress mitigation, and maintenance of auxin transport. Here we provide an overview of the current understanding of how PGB1 biochemistry, localization, and regulatory architecture are connected and of relevance for hypoxia acclimation. We highlight key unanswered questions in our understanding of PGB1 biology that will be essential for clarifying its contribution to hypoxia acclimation and plant environmental resilience.

## Teaser

Phytoglobin 1 acts as a central hub linking hypoxia signaling with nitric oxide turnover and redox–metabolic regulation during plant developmental and stress responses.

## Introduction

Plants occasionally deal with low oxygen conditions (hypoxia) during their life cycle. Hypoxia arises when oxygen demand exceeds supply and can occur natively in plant tissues during development or due to external factors. Chronic development-associated hypoxia has been observed in many plant tissues (Loreti and Perata, 2020; Weits *et al*., 2021) including: germinating seeds (Rolletschek *et al*., 2025), meristematic tissue (Weits *et al*., 2019, 2021), phloem (van Dongen *et al*., 2003), developing leaves (Panicucci *et al*., 2025, Preprint), anthers (Kelliher and Walbot, 2014), pollen (Tadege and Kuhlemeier, 1997), lateral root (LR) initials (Shukla *et al*., 2019; Eysholdt-Derzsó *et al*., 2024), root nodules (Pucciariello *et al*., 2019), and tubers (Geigenberger *et al*., 2000). External environmental factors that cause hypoxia include high altitudes (Abbas *et al*., 2022), flooding (Vashisht *et al*., 2011), nematode infection (Cabello *et al*., 2014), fungal pathogenesis (Chung and Lee, 2020), or darkness (van Veen *et al*., 2016; Triozzi *et al*., 2024). Hypoxia is thus an important determinant of plant development and stress responses.

Plant responses to hypoxia involve various molecular mechanisms that permit sensing and response to fluctuations in oxygen levels (van Dongen and Licausi, 2015). A class of proteins that have emerged as important regulators in this regard are phytoglobins (PGBs), in particular the non-symbiotic class I PGBs (PGB1s). The relevance of PGB1a for hypoxia acclimation is associated with their functions in supporting alternative energy production, oxygen and nitric oxide (NO) signaling, and their integration with key signaling pathways. In this review, we provide an update of recent advancements in our understanding of PGB1 function in the context of hypoxia. This includes current knowledge of PGB1 structure and diversity, *PGB1* expression profiles, and the interconnected physiological and biochemical functions that make it vital for plant oxygen sensing and signaling in various developmental and environmental contexts.

## Hypoxia sensing and signaling

The negative consequences of hypoxia are primarily related to the impact on efficient aerobic energy generation and the oxidative stress associated with reoxygenation (Yeung *et al*., 2018; Sasidharan *et al*., 2021). Oxygen deficit necessitates a shift towards anaerobic energy production through fermentation or alternative mitochondrial pathways. However, these processes are far less efficient than aerobic respiration and are often accompanied by altered mitochondrial function and accumulation of harmful metabolic by-products (van Veen *et al*., 2024). Hypoxia and subsequent reoxygenation can also cause buildup of reactive oxygen species (ROS) and NO, and cytosolic acidification (Felle, 2005; Blokhina and Fagerstedt, 2010; Pucciariello and Perata, 2021). Managing the negative consequences of hypoxia requires plants to actively monitor intracellular oxygen and respond accordingly. Broadly, plants deal with hypoxia through avoidance or coping strategies (Bailey-Serres and Voesenek, 2008). Avoidance responses include escape traits such as aerenchyma formation and/or enhanced shoot elongation, while coping strategies typically involve a quiescence strategy to conserve existing energy reserves via metabolic readjustment or growth suppression (Bailey-Serres *et al*., 2012).

Oxygen sensing in plants is mediated by the oxygen-dependent N-degron pathway which couples protein stability to intracellular oxygen concentration. In Arabidopsis, hypoxia acclimation in the context of environmental stress is mostly associated with the N-degron substrates ETHYLENE RESPONSE FACTOR VII (ERF-VII) transcription factors (TFs), comprising HRE1, HRE2, RAP2.2, RAP2.3, and RAP2.12 (Licausi *et al*., 2011; Giuntoli and Perata, 2018; Manrique-Gil *et al*., 2021; Lavilla-Puerta *et al*., 2023; Zubrycka *et al*., 2023). Additional substrates including VERNALIZATION (VRN) 2 (Labandera *et al*., 2021) and LITTLE ZIPPER (ZPR) 2 (Weits *et al*., 2019) illustrate how low-oxygen sensing intersects with developmental regulation. More potential targets of this N-degron pathway probably exist. All these substrates share a conserved N-terminal MC motif, in which the cysteine residues become a target for PLANT CYSTEINE OXIDASES (PCOs) following methionine removal under oxygen-replete conditions. This is followed by arginylation by ARGINYLTRANSFERASES (ATEs) and ubiquitylation by the E3 ubiquitin-protein ligase PROTEOLYSIS6 (PRT6), and culminating in proteasomal degradation. Hypoxic conditions inhibit this oxygen-dependent process, thus resulting in stable MC proteins.

There is evidence that NO removal (even under normoxic conditions) also results in N-degron pathway substrate (ERF-VII) stabilization, although the exact mode of action remains uncharacterized (Gibbs *et al*., 2014). Thus, NO signaling intersects with oxygen sensing, allowing for additional modulation of plant hypoxia response by other molecular pathways. NO removal capacity is provided by PGBs, of which the hypoxia-inducible PGB1 is of special relevance due to its demonstrated role in NO, oxygen, and redox metabolism (Hill *et al*., 2023; Bykova and Igamberdiev, 2025) and could thus occupy a central position in the regulation of this multilayered oxygen sensing system.

## Phytoglobins—structure and diversity

PGBs are a class of proteins characterized by a heme cofactor, which confers the ability to bind oxygen or other similar ligands with an affinity that depends on the properties of the specific protein. PGBs have long been associated with plant responses to hypoxia and tolerance (Taylor *et al*., 1994; Trevaskis *et al*., 1997; Dordas *et al*., 2003a), including roles in enhancing hypoxia survival (Hunt *et al*., 2002; Dordas *et al*., 2003a), and regulating NO homeostasis (Perazzolli *et al*., 2004; Hebelstrup and Jensen, 2008). Recent studies have firmly established them as central modulators of oxygen sensing and NO signaling (Hartman *et al*., 2019; Mira *et al*., 2021; Liu *et al*., 2022; Hill *et al*., 2023).

Across land plants, PGBs fall into three major classes, as reviewed in Becana *et al*. (2020). PGB1s are the most extensively studied, especially in the context of abiotic stress and hypoxia. Class II PGBs (PGB2s) are similar in structure but are biochemically slightly distinct, less well studied, and appear to be more involved in development (Hunt *et al*., 2001), such as somatic embryogenesis (Mira *et al*., 2023a) or shoot organogenesis (Wang *et al*., 2011). These are also absent in monocots (Becana *et al*., 2020). Symbiotic PGBs (symPGBs) are required in root nodules to maintain micro-oxic environments (Wittenberg *et al*., 1974) and are in most cases related to PGB2s (Trevaskis *et al*., 1997). The class III PGBs (PGB3s) are a part of the structurally distinct truncated globins and remain poorly characterized (Watts *et al*., 2001). Yet, recent research hints towards a role in maintaining hypoxia in tissues (Cheng *et al*., 2025). The distinct expression patterns, mutant phenotypes, and biochemical properties of the different PGB classes indicate specialized roles in plants (Hebelstrup and Jensen, 2008; Hebelstrup *et al*., 2012; Mot *et al*., 2018; Hartman *et al*., 2019).

From an evolutionary perspective, the biochemical activity of PGBs varies among the classes (Becana *et al*., 2020). Intracellular conditions can also significantly impact their enzymatic activity. Consequently, different physiological contexts might require specific PGBs with different biochemical properties. PGBs can have differential affinities for ligands such as oxygen and NO, depending on the organization of the binding pocket. Depending on whether the distal amino acid residue occupies the sixth heme-binding position, PGBs can be hexacoordinate or pentacoordinate. This distal amino acid residue is often a histidine (Smagghe *et al*., 2009), and it competes directly with the ligand for binding the heme iron (Dordas *et al*., 2003b). Thus, distal histidine affinity for the heme iron affects the ligand binding kinetics of the PGBs (Smagghe *et al*., 2009). The stronger the affinity of the distal histidine for the heme, the stronger the hexacoordination, and the lower the affinity of the PGB for oxygen. As PGB1 is weakly hexacoordinate, it has a higher oxygen affinity than the stronger hexacoordinate PGB2, which also results in greater reactivity to NO (Smagghe *et al*., 2009). In contrast, the pentacoordinate PGB3 possesses unexpectedly low oxygen affinity (Watts *et al*., 2001), probably due to active site accessibility (Mukhi *et al*., 2016). Finally, symPGBs are also pentacoordinate, which is thought to be essential for buffering oxygen in hypoxic root nodules (Wittenberg *et al*., 1974). Beyond coordination state, PGB–heme interactions can impact the iron redox and oxidation state, its reactivity with NO, and electron transfer behavior (Astegno *et al*., 2020; Freindorf *et al*., 2024). Ligand binding is also affected by PGB protonation states in the binding pocket, which vary with pH (Mot *et al*., 2018). Additionally, other reactive amino acid residues manage enzymatic activity (Christensen *et al*., 2022). These biochemical properties could thus be highly determinant of PGB functional specialization across tissues and stress conditions (Becana *et al*., 2020).

In Arabidopsis the three non-symbiotic PGBs (PGB1, 2, and 3) have distinct expression patterns reflecting their varying physiological roles. AtPGB1 [AT2G16060, also known as AHB1, HB1, or GLB1 (Hill *et al*., 2016)] is the only member strongly induced by hypoxia and flooding (Hunt *et al*., 2001; Hebelstrup *et al*., 2012). It is one of the 51 core hypoxia genes that are up-regulated independently of tissue type or species (Mustroph *et al*., 2010; Renziehausen *et al*., 2025). Additionally, *PGB1* expression is induced by NO treatments in multiple plant species (Ohwaki *et al*., 2005; Qu *et al*., 2006; Chammakhi *et al*., 2025), and is reduced by overexpression of the NO-scavenging *PGB2* in Arabidopsis (Wang *et al*., 2011). PGB1 is important for stress-related ROS management (Bahmani *et al*., 2019; Liu *et al*., 2022) and is therefore tightly associated with interlinked hypoxia, NO, and ROS. *PGB2* and *PGB3* expression tends to be either constitutive or developmentally regulated, suggesting roles beyond stress responses. These distinct expression profiles highlight functional specialization amongst the PGBs but also mark PGB1 as particularly relevant for stress and hypoxia signaling.

## PGB1 function and properties relevant to hypoxia acclimation

### Influence on intracellular oxygen and NO dynamics

The main action of PGB1 is through binding oxygen, which can occur only when the PGB1 heme iron is in a ferrous state (Fe^2+^). PGB1(Fe^2+^)–O_2_ can react with NO to form nitrate and ferric PGB1(Fe^3+^), which is an NO dioxygenation reaction (Perazzolli *et al*., 2004). This is the basis for most of our understanding of its biological relevance.

Under hypoxic conditions, this NO dioxygenation reaction can form an important link in the low-oxygen metabolic chain, called the PGB–NO cycle. Multiple cellular processes can contribute to NO production under these conditions. NO is generated by nitrite reductase (NiR) activity or as a mitochondrial byproduct (Gupta *et al*., 2005; Wany *et al*., 2019). PGB1 subsequently recycles the NO back to nitrate (Igamberdiev *et al*., 2005). The ferric PGB1(Fe^3+^) is reduced back to ferrous PGB1(Fe^2+^) by as yet unidentified electron donors, closing the cycle and regenerating the NAD^+^ pool. This recycled NAD^+^ can again be used for glycolysis. The PGB–NO cycle can thus be used by the plant to generate small amounts of ATP, without some of the negative side effects of alternative hypoxic pathways (Igamberdiev and Hill, 2004). The relatively high affinity of PGB1 for oxygen may ensure consistent binding even under low-oxygen conditions for stable energy production.

During hypoxic or fully anoxic conditions, deoxygenated PGB(Fe^2+^) can itself produce NO from nitrite (Sturms *et al*., 2011; Tiso *et al*., 2012; Kumar *et al*., 2016). This consumes one proton (Igamberdiev *et al*., 2010; Sturms *et al*., 2011; Tiso *et al*., 2012), suggesting that PGB1 activity could mitigate intracellular acidification during hypoxia. PGB1 has greater NO-generating activity than PGB2 or PGB3 (Zagrean-Tuza *et al*., 2024), which could imply a specific biological function during anoxia. Indeed, *PGB1* is induced by hypoxia and nitrite or nitrate supplementation (Ohwaki *et al*., 2005), and NO appears essential for hypoxia acclimation (Sasidharan *et al*., 2018; Armstrong *et al*., 2019). The influence of PGB1 in determining the pools of NO versus more oxidized soluble nitrogen compounds such as nitrite and nitrate could be vital in hypoxia sensing and response.

### Effects on cellular redox, nitrosylation, and ROS interplay

The influence of PGB1 on cellular NO levels can impact plant performance due to the significant but complex role of NO in both plant development and stress response (Liu *et al*., 2024). NO can be produced through enzymatic reactions or as a mitochondrial byproduct (Singh *et al*., 2020; Hill *et al*., 2023). As a reactive signaling molecule, NO influences transcription, protein stability, and metabolism through several reversible post-translational modifications of proteins, which include cysteine *S*-nitrosylation and tyrosine nitration (Bogdan, 2001; Grün *et al*., 2006). Many protein substrates are known to be functionally affected by *S*-nitrosylation, which significantly impacts plant stress responses (Feng *et al*., 2019). While PGB1 itself can also be *S*-nitrosylated (Perazzolli *et al*., 2004), this does not affect its *in vivo* biochemical functioning (Rubio *et al*., 2019). Thus, PGB1, through NO, can integrate many different hormonal pathways (Ergashev *et al*., 2025), and can connect them to hypoxia signaling via ERF-VII stability (Gibbs *et al*., 2014).

During plant stress responses, NO can, for example, induce programmed cell death (Stasolla and Hill, 2017), regulate actin (Kasprowicz *et al*., 2009) and microtubule organization (Shi *et al*., 2009; Krasylenko *et al*., 2012), or regulate abscisic acid (ABA)-directed and Ca^2+^-mediated stomatal conductance (Garcia-Mata *et al*., 2003; Guo *et al*., 2003; Rubio *et al*., 2019). In addition, NO is key during developmental decisions, by repressing growth, flowering, and bolting (Simpson, 2005), inducing leaf hydathode senescence (Hebelstrup *et al*., 2006), inducing xylem (Gabaldón *et al*., 2005) and *in vitro* tracheary element differentiation (Mira *et al*., 2019), and can affect stomatal development (Fu *et al*., 2016; Zhou *et al*., 2024). Thus, by altering the NO response equilibrium (Hebelstrup and Jensen, 2008), PGB1 could play a pivotal role in tuning plant responses to developmental cues and environmental stresses.

Like NO, ROS are redox-active molecules widely implicated in plant stress response and development (Mittler *et al*., 2022). A broad range of conditions can trigger molecular processes that generate ROS, which can function as both signaling molecules and agents of oxidative stress. ROS can alter cellular redox states and protein properties and thereby relay signals to elicit a plant response. Like NO, ROS affect protein function by oxidative post-translational modification of cysteine and methionine residues (Waszczak *et al*., 2018). Alternatively, stress conditions can cause photosynthesis or accumulation of respiration-related ROS which are harmful for the plant.

Oxidative stress management is key to hypoxia survival, and PGB1 plays a significant role in reducing ROS and related damage (Sainz *et al*., 2013; Bahmani *et al*., 2019; Liu *et al*., 2022). Limiting ROS production in a variety of circumstances might involve the modulation of respiratory burst oxidase homolog (*RBOH*) gene expression by PGB1 (Youssef *et al*., 2016; Hammond *et al*., 2020; Mira *et al*., 2021). It is unclear how this regulation is established, but NO is upstream of ROS in a positive feedback loop (El-Shetehy *et al*., 2015), and alternatively some RBOH enzymes have reduced activity upon *S*-nitrosylation (Yun *et al*., 2011). In *Brassica napus*, *PGB1* overexpression enhanced the expression of antioxidant genes (El-Khateeb *et al*., 2023). PGB1-mediated oxidative stress tolerance could be of functional relevance, for example post-submergence, where reoxygenation leads to excessive ROS production, causing additional severe oxidative stress, photoinhibition, desiccation, and starvation (Yeung *et al*., 2019). Additionally, PGB1-mediated NO management enhances the capacity of plants to deal with heavy-metal-related ROS stress (Bahmani *et al*., 2019). Thus, by impacting NO levels, PGB1 could also fine-tune ROS levels, but details of this interaction remain to be elucidated.

### Post-transcriptional regulation, oligomerization state, and subcellular localization

Post-transcriptional regulation, protein interactions, oligomeric state, and subcellular localization further shape PGB1 function. In Arabidopsis seedlings exposed to ethylene and subsequent hypoxia stress, PGB1 protein and mRNA levels strongly correlated (Liu *et al*., 2022), suggesting little translational and post-translational regulation. However, evidence for similar associations is missing for other conditions or plant species, and other mechanisms could potentially play a role. For example, in the Arabidopsis *C24* accession, *pPGB1:β-glucuronidase* (*GUS*) reporter lines were only hypoxia responsive if the 3′-untranslated region (UTR) was included (Hunt *et al*., 2001). The legume *Medicago truncatula PGB1-2* is subject to environment-dependent alternative splicing to generate four isoforms, some of which are larger than other known hemoglobins across kingdoms and contain multiple heme groups (Villar *et al*., 2020). Finally, PGB1 itself is also subjected to ubiquitination and proteasomal degradation, which can also affect its stability (Inès *et al*., 2025).

PGB1 exists in a concentration-dependent equilibrium between monomeric and dimeric states, with increased dimerization at elevated protein levels (Astegno *et al*., 2020). This PGB1 dimerization (Bogusz *et al*., 1988) impacts its biochemical activity through allosteric interactions (Couture *et al*., 1999; Eaton *et al*., 1999; Astegno *et al*., 2020). Dimerization occurs through a hydrophobic residue cluster in the dimerization domain (Christensen *et al*., 2023), which shifts PGB1 heme coordination from hexa- to pentacoordinate, thereby increasing the affinity for ligands such as oxygen (Astegno *et al*., 2020). Oxygen binding increases PGB1 dimer stability, creating cooperative binding behavior (Mukhi *et al*., 2013). Thus, the NO-scavenging capacity of PGB1 is expected to rise more sharply than its protein levels when induced by environmental or developmental factors. This enhanced NO turnover is not the case for PGB2 (and symPGBs) (Astegno *et al*., 2020), suggesting distinct molecular functions and highlighting the unique role of PGB1 in integrating oxygen and NO signaling. Future research should aim to confirm the functionality of this conserved dimerization interface in an *in vivo* setting. Overall, integrating PGB biochemical properties with their function and regulation through *in vivo* studies could prove vital for developing more stress-resilient plants.

Some studies have revealed the intracellular localization of PGB1 through use of antibody staining or protein–reporter fusions. These show that PGB1 can be localized in the cytoplasm (Rubio *et al*., 2019; Mira *et al*., 2023b), nucleus (Seregélyes *et al*., 2000; Rubio *et al*., 2019; Mira *et al*., 2023b), or in plastids: chloroplasts in the shoot, and amyloplasts in the root (Rubio *et al*., 2019). The precise subcellular localization of PGB1 could indicate involvement in distinct subcellular pathways. The specific function that PGB1 fulfills could logically depend on its localization. In association with mitochondria in the cytoplasm, PGB1 could mediate alternative energy generation through the PGB1–NO cycle, whereas in plastids it could aid detoxification, and in the nucleus it could assume a role in signaling. Subcellular localization appears to be functional for PGB2, which only functions in somatic embryogenesis if present in the nucleus (Godee *et al*., 2017). Nuclear function of PGBs is hypothesized to be mediated through flavin-dependent reduction of their heme groups (Sainz *et al*., 2013). During hypoxia, PGB1 seems to shift from the nucleus to the cytoplasm (Mira *et al*., 2024). During drought stress in *B. napus*, PGB1 appears predominantly in the cytoplasm (Mira *et al*., 2023d), suggesting stress-dependent localization, although higher resolution imaging might be required to ascertain this. Whether subcellular localization also impacts PGB1 functioning *in planta* remains to be determined, but would be valuable to uncover its role in hypoxia acclimation and signaling.

## Phytoglobins in developmental and environmental hypoxia

### Plant development

Hypoxia is a recurring feature of plant development, arising in tissues where anatomy or metabolic activity reduce oxygen supply and/or enhance demand. Studies with reporter lines and antibodies have revealed that tissue-specific PGB1 expression patterns coincide with the presence of hypoxic niches in the plant (Fig. 1). In the root, *PGB1* is expressed strongly in the LR cap (Hunt *et al*., 2001; Zhao *et al*., 2008; Mira *et al*., 2023b), vasculature (Hunt *et al*., 2001; Zhao *et al*., 2008), and the epidermis (Mira *et al*., 2023b, 2024). The vasculature is hypoxic due to high tissue density, surrounding tissue barriers, and high metabolic activity (Colmer *et al*., 2020; Jiménez *et al*., 2026). Older, thicker parts of the root are more hypoxic, which is in accordance with elevated *PGB1* expression at the thick root–shoot junction observed in some studies (Bahmani *et al*., 2019). The association with hypoxic niches for other *PGB1*-expressing tissues at the root surface such as the LR cap and epidermis, however, remain unclear. Still, soils can rapidly become hypoxic during flooding (Vashisht *et al*., 2011), necessitating constitutive *PGB1* expression for sensing and signaling in these root tissues. Indeed, the ability of the root to avoid hypoxic soil patches by regulating ERF-VII-dependent and auxin-mediated root bending (Eysholdt-Derzsó and Sauter, 2017) appears to be regulated by PGB1 (Mira *et al*., 2024). Despite the importance of PGB1 for sustaining stem cell niche viability under stress (Mira *et al*., 2017, 2018, 2020, 2023b; Hill *et al*., 2023), its expression in the quiescent center (QC) remains unresolved (Hill *et al*., 2026). As root differentiation is regulated by NO and ROS (Mukherjee and Corpas, 2023), it is likely that PGB1 plays a central role in orchestrating its development during physiological or stress conditions.

**Fig. 1.**
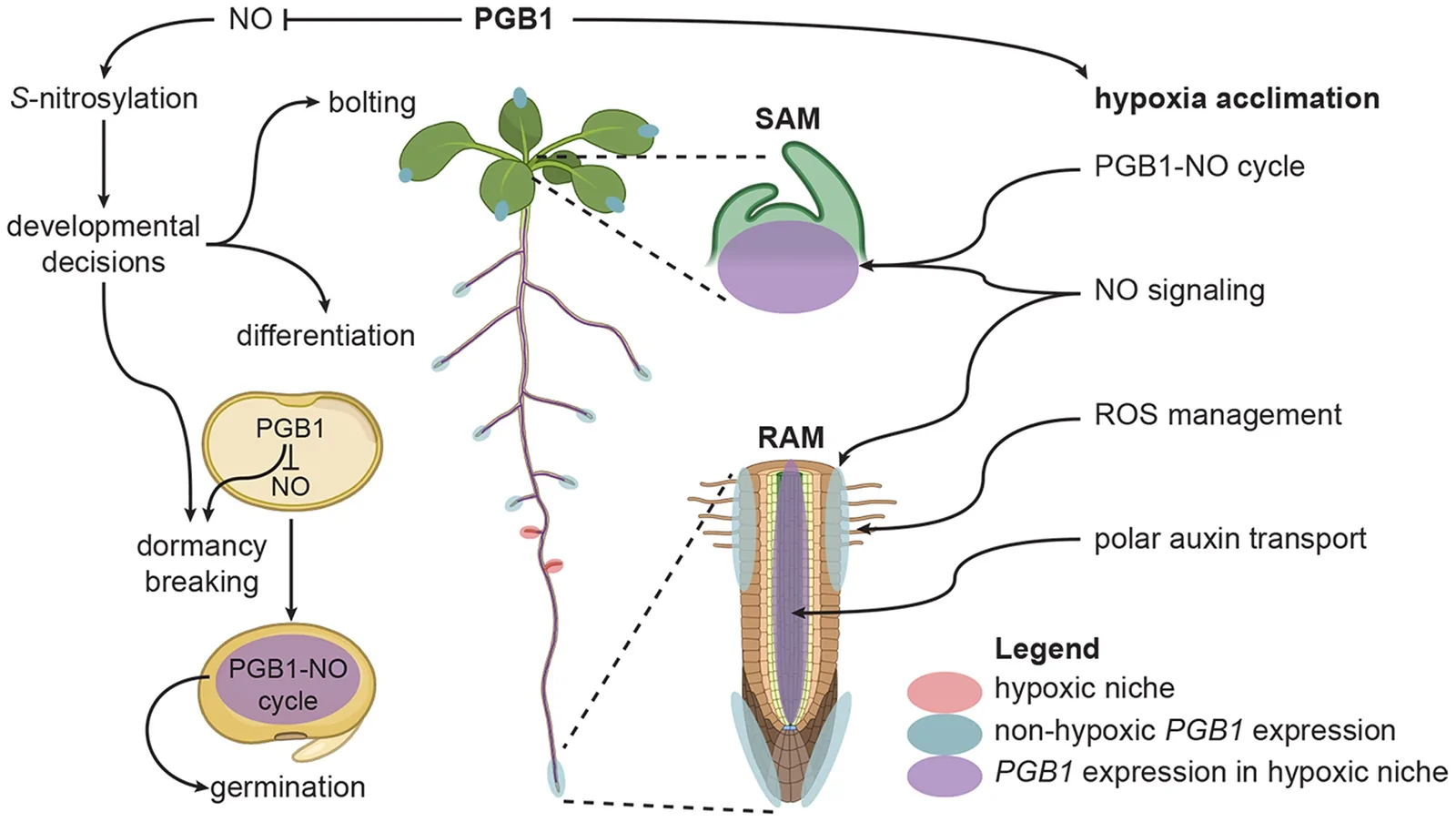
Phytoglobin 1 (PGB1) function and expression are linked to native hypoxic niches in the plant. *PGB1* expression largely overlaps with developmental hypoxic niches (purple ovals), with some exceptions (red ovals), and also occurs in oxygen-replete tissues (blue ovals). An important function of PGB1 is in nitric oxide (NO) turnover. By affecting NO-dependent protein *S-*nitrosylation, PGB1 influences developmental decisions in the plant life cycle. In hypoxic niches, PGB1 maintains energy generation through the PGB1–NO cycle. Management of NO and ROS by PGB1 aids in plant stress tolerance and signaling, and maintenance of polar auxin transport. Subcellular localization of PGB1 could potentially be linked to its distinct biological roles.

LR development is another hypoxia-regulated process, where auxin, ABA, and ERF-VII TFs interact to determine root quiescence or growth (Shukla *et al*., 2019; Eysholdt-Derzsó *et al*., 2024). During the process of LR emergence and maturation, oxygen availability becomes increasingly limited as cell divisions intensify. However, *PGB1* expression is induced only during later stages of LR development, making it unlikely that it plays a significant role during this process. Surprisingly, *PGB1* overexpression increased LR number (Hunt *et al*., 2002), hinting at the ability of PGB1 to steer ERF-VII-dependent processes to regulate developmental programs, which could prove vital for stress-responsive morphological acclimation.

In the shoot, *PGB1* expression in the vasculature is continuous with that of the root (Hunt *et al*., 2001; Zhao *et al*., 2008; Triozzi *et al*., 2024), which can also be hypoxic (van Dongen *et al*., 2003). The strongest *PGB1* expression occurs in the youngest developing tissues such as the shoot apical meristem (SAM) and young emerging leaves (Hebelstrup and Jensen, 2008; Triozzi *et al*., 2024). This expression pattern aligns well with the known hypoxic niche associated with high metabolic demand in these rapidly developing tissues (Weits *et al*., 2021). Similarly, during the night, fast growing plant tissues become hypoxic and display signature hypoxic gene expression, including *PGB1* (Triozzi *et al*., 2024). This cyclic-hypoxia-related *PGB1* expression suggests its requirement during hypoxia derived from high metabolic activity, when mitochondrial capacity becomes limiting. This is linked to PGB1 being part of the core mitochondrial retrograde response (Eysholdt-Derzsó *et al*., 2023). Likewise, decreased carbon availability reduces *PGB1* expression (Nie and Hill, 1997), possibly through limiting oxygen consumption. Fast growing tissues might therefore require PGB1–NO cycle activity for alternative respiration. This is in accordance with the induction of *PGB1* in germinating seeds (Barreto *et al*., 2022; Nie *et al*., 2022; Sousa *et al*., 2025), which can also experience hypoxia due to high metabolic activity and limited oxygen availability (Rolletschek *et al*., 2025).

PGB1 is also present in cotyledons and mature leaves (Hebelstrup *et al*., 2006; Hebelstrup and Jensen, 2008; Rubio *et al*., 2019; Triozzi *et al*., 2024), leaf hydathodes (Hebelstrup *et al*., 2006; Hebelstrup and Jensen, 2008; Triozzi *et al*., 2024), the hypocotyl (Hunt *et al*., 2001; Zhao *et al*., 2008), and developing flower buds (Hebelstrup *et al*., 2006). Strong expression in developing flower buds may also reflect a function in hypoxia acclimation due to elevated energy requirements, whereas expression in hydathodes and leaves could be related to functions distinct from hypoxia, such as oxidative stress mitigation in photosynthesizing cells, or environment-derived NO signal integration.

PGB1 is also vital for developmental pathways in the context of hypoxia that require a tight NO balance, such as the decisions underlying differentiation or germination. In *M. trunculata*, PGB1-1 regulates nodulation through strict NO control (Berger *et al*., 2020). For seed dormancy breaking, PGB1 plays a vital role as inadequate NO balance, ROS levels, or redox state lead to the inability to initiate germination (Bykova and Igamberdiev, 2025). During later stages of germination, PGB1 is required for hypoxic metabolism and can even enhance germination rates under adverse conditions (Cochrane *et al*., 2017; Zafari *et al*., 2020). *PGB1* expression regulation is required for transition to reproductive developmental programs such as bolting (Hebelstrup and Jensen, 2008) or flower development (Hebelstrup *et al*., 2006), or for proper development of leaves. During stress responses, PGB1 is vital for maintaining an undifferentiated state of root meristem cells (Hill *et al*., 2023), but no effects of down-regulating *PGB1* have been observed under standard conditions (Mira *et al*., 2023b). Therefore, PGB1 functioning is likely to be related to maintaining energy balance in tissues with native hypoxia, while steering development or steering signaling in the non-hypoxic tissues in contact with the environment.

### Flood acclimation

In flooded plants, gas exchange with the environment is impeded since water forms a significant barrier to gas diffusion. As a result, flooded tissues experience changes in the dynamics of gases such as oxygen and carbon dioxide. The imposition of hypoxia depends on the conditions of flooding. In waterlogged soils, oxygen depletion occurs rapidly, resulting in hypoxic conditions for only plant roots. If the entire plant is submerged, hypoxic conditions can also occur in the shoot. However, the speed of hypoxia onset and severity will depend on the light-dependent photosynthetic capacity of the shoots. In addition, flooded tissues will experience accumulation of the volatile hormone ethylene due to physical entrapment (Voesenek *et al*., 1993). Flooding also impacts the redox status of the plant, including changes in NO and ROS dynamics. Upon reoxygenation, oxidative stress is a major stressor associated with excessive ROS production (Yeung *et al*., 2019). PGB1 is impacted by many of these stress signals at different regulatory levels but itself can also influence stress signal dynamics. PGB1 is thus a critical component during flooding/hypoxia stress acclimation embedded in the complex underlying signaling networks. Here we outline, based on current knowledge, the involvement of PGB1 during early, late, and post-flooding phases, and speculate on its multi-faceted role for flood survival.

#### Early flooding

Flooding-induced buildup of ethylene occurs irrespective of light availability, or tissue, or organ, and is proposed to act as an early warning signal to improve tolerance to imminent hypoxia (Voesenek and Sasidharan, 2013; Sasidharan *et al*., 2018; Sasidharan, 2022; Ugalde, 2022).

Accordingly, an ethylene pre-treatment enhances flooding/hypoxia tolerance in several species (van Veen *et al*., 2013; Hartman *et al*., 2020). In Arabidopsis, PGB1 has been demonstrated to be an important mediator of this ethylene effect as *pgb1-1* mutants fail to show this beneficial effect (Hartman *et al*., 2019). Ethylene significantly up-regulates both PGB1 transcript (Heckmann *et al*., 2006; Hartman *et al*., 2019, 2020; Liu *et al*., 2022) and protein levels under normoxic conditions (Liu *et al*., 2022). Although the nature of this induction remains unclear, the effect of PGB1 during this pre-hypoxia ethylene-mediated acclimation period is related to its NO-scavenging ability and consequent stabilization of ERF-VII TFs (Hartman *et al*., 2019). Interestingly, while ethylene-mediated ERF-VII stabilization was observed during normoxic conditions, target gene activation only occurred upon hypoxia. It currently remains unknown how NO functions in the PRT6 N-degron pathway (Gibbs *et al*., 2014) and why NO removal stabilizes ERF-VIIs, but the central role of PGB1 in ethylene-mediated hypoxia acclimation is evident.

#### Prolonged flooding expression regulation

After the initial flooding phase that allows for ethylene-mediated acclimation, hypoxia onset will occur. The ERF-VII TFs regulate the transcriptomic changes required for the hypoxia acclimation response via activation of downstream signaling pathways including hypoxia-responsive promoter element- (HRPE) containing target genes (Mustroph *et al*., 2009; Gasch *et al*., 2016). These core hypoxia genes include *PGB1* and, in this phase, mRNA expression levels increase even further. The induction of *PGB1* by hypoxia is stronger than that by ethylene, but the ethylene pre-treatment preceding hypoxia onset enhances hypoxic PGB1 induction. This could be due to the positive feedback loop of PGB1-mediated ERF-VII stabilization (Fig. 2) (Hartman *et al*., 2019; Liu *et al*., 2022). Elements of this regulatory loop are conserved in other species, such as *Brachypodium distachyon*, except that one of the ERF-VIIs also reduces *PGB1* expression (Hsiao *et al*., 2024). Additionally, ERF-VII-dependent transcriptional regulation is attenuated by MEDIATOR SUBUNIT25 (MED25), yet only RAP2.2 and RAP2.12 interact with MED25 (Schippers *et al*., 2024). The fact that hypoxic *PGB1* induction is not affected by MED25 regulation suggests that not all ERF-VIIs regulate *PGB1* in equal measure. Also, RAP2.3 is involved in ethylene signaling (Kim *et al*., 2018), which could further complicate *PGB1* regulation.

**Fig. 2.**
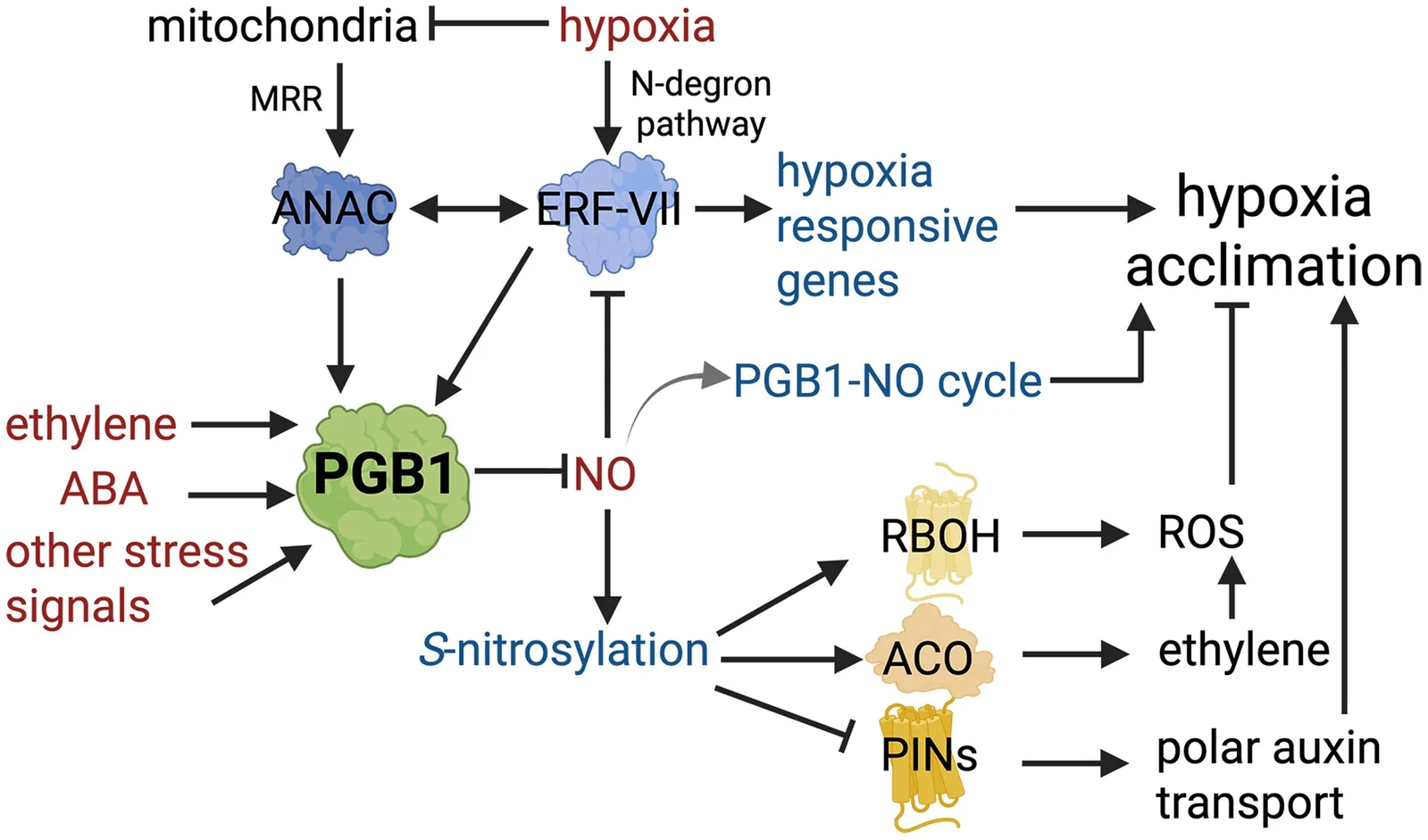
PGB1 is a central hub in a complex signaling and hypoxia acclimation network. PGB1 functions at the crossroads of hypoxia signaling, linking oxygen sensing, nitric oxide dynamics, and redox control to support hypoxia acclimation. Several stress and hormonal cues (red font) and transcription factors (ANAC, ERF-VIIs) can induce PGB1 expression, activating downstream effects (blue font) that eventually contribute to hypoxia acclimation. As the PGB1–NO cycle is derived from the PGB1-mediated NO scavenging rather than a downstream pathway, this connection is indicated with a distinct curved gray arrow. Created in BioRender. Sasidharan, R. (2026) https://BioRender.com/6jfpxep.

Hypoxia reduces mitochondrial functioning as oxygen is required for respiration and triggers mitochondrial retrograde regulation (MRR). MRR during hypoxia-induced mitochondrial dysfunction is mediated by the NO APICAL MERISTEM/ARABIDOPSIS TRANSCRIPTION ACTIVATION FACTOR/CUP-SHAPED COTYLEDON (NAC) TFs that recognize the mitochondrial dysfunction motif (MDM) in promoters of hypoxia-responsive genes (De Clercq *et al*., 2013). ANAC013, ANAC016, and ANAC017 up-regulate *PGB1* through the two MDMs in its promoter, of which the one closest to the *PGB1* transcription start site is vital (Eysholdt-Derzsó *et al*., 2023). Additionally, ANAC013 up-regulates the ERF-VII HRE2, potentially amplifying the response of *PGB1*. During hypoxia, ANAC013 is cleaved off the endoplasmic reticulum membrane by the RHOMBOID-LIKE 2 (RBL2) protease, allowing for rapid hypoxia response (Eysholdt-Derzsó *et al*., 2023). However, it is unknown during which phase of hypoxia ANAC016 and ANAC017 are active, despite ANAC017 providing hypoxia tolerance and its involvement in chloroplast signaling (Van Aken and Pogson, 2017; Meng *et al*., 2020). MRR also stabilizes the ERF-VIIs through UNCOUPLING PROTEIN 1 (UCP1), which acts upstream of the PCO branch of the PRT6 N-degron pathway during salt stress and (developmental) hypoxia (Barreto *et al*., 2022). The fact that UCP1-dependent ERF-VII regulation is required for the expression of *PGB1* suggests that *PGB1* could be at the intersection of multiple hypoxia signaling pathways. In conclusion, several signaling networks appear to mediate the sustained expression of *PGB1* during prolonged flooding, underscoring its requirement and potentially distinct functions for hypoxia acclimation.

#### Prolonged flooding hypoxia acclimation

Since hypoxia itself can stabilize ERF-VIIs, the contribution of PGB1 in this regard is probably not the most vital function. Instead, its bidirectional relationship with NO might be of greater significance. In addition to stabilizing the ERF-VIIs, PGB1 plays a role in maintaining energy levels within the cell through the PGB–NO cycle (Hill *et al*., 2023). During hypoxia, NO can be generated by mitochondria from nitrite as the final electron acceptor in addition to other NO-generating processes (Singh *et al*., 2020; Hill *et al*., 2023). Additionally, ANAC013 and ANAC017 induce the expression of Alternative Oxidase 1 (*AOX1*) (Eysholdt-Derzsó *et al*., 2023), which can facilitate this mitochondrial NO production (Hill *et al*., 2023). The PGB–NO cycle allows for NAD^+^ recycling for glycolysis and moderate ATP generation (Igamberdiev and Hill, 2004). Thus, *PGB1* together with other genes aids the plant in alternative oxidative pathways to alleviate the downsides of hypoxic cellular metabolism. In line with this, nitrate supplements could increase plant hypoxia tolerance, which is mediated through NO (Timilsina *et al*., 2022; Hesari *et al*., 2025), as well as the expression of both *PGB1* and NO production genes, such as *NIA1*, *NIA2*, *NiR* and *AOX1a*, and *ERF-VII* genes (Wang *et al*., 2000; Wany *et al*., 2019; Safavi-Rizi *et al*., 2024). Nitrite reduces or increases *PGB1* expression during normoxia or hypoxia, respectively (Kumari *et al*., 2022). In barley (*Hordeum vulgare* L.), *PGB1* is induced by the inhibition of respiratory pathways (Nie and Hill, 1997), probably mediated by Ca^2+^ signaling (Nie *et al*., 2006), or MRR-related signaling.

However, direct *in vivo* evidence for the functioning of the PGB1–NO cycle remains scarce. NADPH-mediated PGB1(Fe^3+^) reduction to PGB1(Fe^2+^)–O_2_ (Perazzolli *et al*., 2004) is attributed primarily to a monodehydroascorbate reductase (MDHAR) activity (Igamberdiev *et al*., 2006), and has been monitored mostly through indirect methods (Wany *et al*., 2019; Kumari *et al*., 2022). Additionally, mostly organic PGB1 reductants have been reported (Hebelstrup *et al*., 2007; Becana *et al*., 2020; Bykova and Igamberdiev, 2025). Direct interaction of PGB1 with such MDHAR enzymes has not been fully described, but the requirement for MDHAR in PGB1 reduction suggests a vital role for this enzyme in hypoxia acclimation and could even link PGB1 and ascorbate-dependent ROS scavenging.

Still, the effect of PGB1 on cellular metabolism during hypoxia is significant. In *Zea mays*, PGB1 shifts the redox balance toward a more oxidized state (Hebelstrup *et al*., 2007; Mira *et al*., 2020), which is associated with the stem cell-like identity of those cells (Hill *et al*., 2023). In addition, PGB1 reduces *S*-nitrosylation of tricarboxylic acid (TCA) cycle enzymes to increase cycle activity, maintain starch levels, and reduce fermentation (Mira *et al*., 2023c). Thus, PGB1 during flooding-induced hypoxia can contribute to maintaining energy levels in cells lacking full capacity aerobic respiration.

In contrast to its benefits as an early flooding signal mediating hypoxia acclimation, ethylene also accelerates senescence and cell death during flooding-induced hypoxia (Rankenberg *et al*., 2024), contrasting with the role of PGB1 as an inhibitor of these processes (Hill *et al*., 2023). This apparent contradiction can be explained by a temporal regulatory model: *PGB1* is induced during early flooding by ethylene for hypoxia acclimation, but its continued expression in later stages may mitigate the negative effects of ethylene, effectively dampening the ethylene response. NO can modulate ethylene biosynthesis by facilitating *S*-nitrosylation of ACO enzymes, either enhancing (Liu *et al*., 2023) or reducing production depending on the circumstances (Maurya *et al*., 2025). Accordingly, *PGB1* knockdown and *PGB2* knockout lines show elevated NO and ethylene production during waterlogging (Hebelstrup *et al*., 2012). Elevated ethylene can enhance ROS production and eventually cell death (Mira *et al*., 2018). Cells expressing PGB1 are thus potentially protected from ethylene-induced cell death, but high-resolution spatial expression profiling of PGB1 has not yet been explored. It is plausible that the cell death protective role of PGB1 is cell type specific. For example, in *Z. mays*, flooding-induced aerenchyma requires ethylene-induced cell death (Rajhi *et al*., 2011), a process which PGB1 prevents in stem cells (Mira *et al*., 2016). This modulatory effect of PGB1 on ethylene levels and cell death appears conserved under other stresses, such as drought (Mira *et al*., 2017; Montilla-Bascon *et al*., 2025). Thus, ethylene-induced PGB1 expression may serve as a feedback mechanism to limit the long-term detrimental effects of the hormone during flooding.

### Root meristem maintenance

Another important hypoxia-related function of PGB1 during environmental (flooding) contexts, is maintenance of the root apical meristem and QC function (Mira *et al*., 2018, 2023b; Hill *et al*., 2023). This is probably a non-cell-autonomous effect, as PGB1 is not induced in the QC itself during hypoxia (Hill *et al*., 2026). Moreover, this is attributed to the impact of PGB1 on polar auxin transport (PAT), which is essential for root development and environmental sensing (Adamowski and Friml, 2015). The main players in this PAT are the polarly localized PIN-FORMED (PIN) proteins (Blilou *et al*., 2005). These auxin exporters induce a net flow towards the QC, where an auxin maximum maintains stem cell identity and organization. From there, PINs distribute auxin back shootward through the epidermis and cortex, creating a ‘reverse fountain’ of auxin flow. NO can disrupt the localization of PIN proteins, possibly via dysregulated membrane vesicle trafficking (Kasprowicz *et al*., 2009), and under flooding stress can cause a loss of the PAT and stem cell identity (Mira *et al*., 2023b). Additionally, NO affects nuclear auxin response through *S*-nitrosylation of TRANSPORT INHIBITOR RESPONSE1 (TIR1)/AUXIN-SIGNALING F-BOX (AFB) (TIR1/AFB) proteins that mediate transcriptional auxin response (Terrile *et al*., 2012). This consequently can lead to root senescence and eventually death, which can be mitigated by PGB1-mediated NO scavenging to maintain PIN localization, QC auxin maximum, and meristem functioning (Mira *et al*., 2023b). Recent developments indicate that this process is mediated through the ERF-VII TFs (Mira *et al*., 2024), yet the effect of this pathway is limited to stress-related conditions.

An intriguing question is how PGB1 regulation is achieved to precisely stabilize PINs where needed. Early studies have indicated that PGB1 is not auxin induced (Hunt *et al*., 2001; Goda *et al*., 2004), despite the presence of a few single auxin response elements (AuxREs) in its promoter (Stasolla *et al*., 2019). However, these AuxREs are not in configurations that are more strongly auxin responsive (Freire-Rios *et al*., 2020). Still, the vasculature is a hypoxic tissue with high physiological *PGB1* expression, and therefore the function of PGB1 in auxin signaling could be hypoxia related.

### Crosstalk with other environmental stresses

Besides hypoxia, PGB1 is also implicated in improving plant resilience to other stresses. Notably, its roles in these stresses have many parallels to hypoxia, highlighting the interconnectedness of stress signaling pathways and positioning of PGB1 as a central regulatory hub. In drought-stressed plants, PGB1 aids survival by sustaining PAT (Mira *et al*., 2017, 2023d; Hammond *et al*., 2020) and reducing ethylene production (Hammond *et al*., 2020; Montilla-Bascon *et al*., 2025) to prevent cell death. Resistance to heavy metal stress is mediated through PGB1-derived reductions in NO and ROS (Bahmani *et al*., 2019). It appears that drought or osmotic stress can also lead to hypoxia in plant roots, where the PGB1–NO cycle assists in energy generation (Chammakhi *et al*., 2022). ABA can induce PGB1, which in stomata scavenges NO to increase stomatal closure and reduce water loss (Rubio *et al*., 2019). Conversely, ABA has also been shown to repress *PGB1* expression in barley aleurone (Nie *et al*., 2022), and PGB1 can in turn impact ABA production (Kapoor *et al*., 2018), highlighting a bidirectional relationship between PGB1 and ABA signaling. Indeed, also during seed germination, many factors interact with PGB1, NO, gibberellic acid (GA), ABA, and ethylene signaling among others to regulate dormancy breaking (Bykova and Igamberdiev, 2025). Any process where PGB1 reduces cellular NO can lead to ERF-VII-dependent hypoxia signaling, and any process in which hypoxia signaling is involved, PGB1 could be implicated. For example, ERF-VIIs and thus potentially PGB1 could be involved in pathogen response (García *et al*., 2024). Many other conditions can induce *PGB1* and simultaneously generate NO (Hill, 2012). Thus, an open question is whether regulation of PGB1 by other hormones, such as ethylene (Hartman *et al*., 2019), could also play a role in hypoxia-related processes or stresses, especially when these are closely linked.

## Conclusion

PGB1 clearly occupies a central position at the intersection of oxygen sensing, NO homeostasis, redox control, and hormonal pathways. Its biochemical activity and regulatory architecture enable it to integrate multiple environmental and developmental signals into coordinated physiological outputs. It is especially tightly connected to hypoxia acclimation, reflected by its expression in developmental hypoxic niches (Fig. 1), and stress response (Fig. 2). Despite our current understanding of the broad biological function of PGB1, from NO scavenging and ROS mitigation to energy generation, signaling, and cell fate decisions (Stasolla *et al*., 2019; Hill *et al*., 2023), many questions remain.

It remains unclear which parts of the spectrum of PGB1 molecular functions are vital in various environmental or developmental contexts. The occurrence of multiple PGB1s in some species suggests functional specialization between members (Berger *et al*., 2020; Villar *et al*., 2020; Esquinas-Ariza *et al*., 2026). Another important but underexplored aspect is the relevance of PGB1 subcellular localization, despite some initial reports on PGB2 (Godee *et al*., 2017). How spatial distribution relates to distinct molecular functions, and if and how developmental and environmental cues dynamically regulate PGB1 localization require further investigation. Also requiring further probing is how post-translational modifications and protein–protein interactions impact PGB1 reactivity. The biochemical properties of PGB1 have been revealed by in-depth analyses of its *in silico* functionality, yet validation via *in vivo* studies is missing. Temporal and cell-type-specific *PGB1* expression profiles must be documented using high resolution reporter lines and imaging techniques, to discern how its site of action connects with biological function. Similarly, studies on the *cis*-regulatory landscape of the *PGB1* promoter would allow untangling of its complex regulation by many external and endogenous cues. This would facilitate tissue- or stress-specific tailoring of PGB1 for improved stress tolerance or development of plants. Such questions could be addressed using cell-type-specific gain- or loss-of-function mutants of PGB1.

Most PGB1 research in Arabidopsis has relied on the use of a limited set of transgenic lines—two *PGB1* overexpression and two knockdown mutants (Perazzolli *et al*., 2004; Hebelstrup *et al*., 2006; Hebelstrup and Jensen, 2008), which could introduce unintended biases due to transgene insertion site effects. Still, the effect of PGB1 on NO scavenging or hypoxia tolerance appears to be consistent even across species (Dordas *et al*., 2003a; Perazzolli *et al*., 2004; Hebelstrup *et al*., 2012; Mira *et al*., 2021; El-Khateeb *et al*., 2023). In contrast, the influence of PGB1 on less-characterized processes—such as JA, ABA, or ethylene signaling, and broader aspects of development remain poorly explored. Additionally, antisense knockdown lines could retain residual *PGB1* expression (Hebelstrup *et al*., 2006), highlighting the need to complement these studies with true loss-of-function alleles.

To date, it remains unclear whether complete genomic knockouts of PGB1 are developmentally viable, as only a single genomic knockout line of unknown provenance has been reported (Rubio *et al*., 2019). Consequently, the generation of additional PGB1 mutant and reporter lines would be highly beneficial. With the advent of CRISPR/Cas technologies, diverse types of PGB1 coding sequence and promoter deletion mutants can now be readily produced. Such lines could prove powerful tools to validate many synthetic biology approaches and to dissect regulatory mechanisms. Additionally, combining reporter lines with functional complementation studies would enable rigorous validation of cell-type-specific *PGB1* expression and function. Together, these observations underscore that expanding the genetic toolbox (additional loss- and gain-of-function lines) and mapping direct downstream targets (e.g. via *S*-nitrosylation profiling) will be key to resolving the diverse roles of PGB1.

Lastly, the centrality of PGB1 in the hypoxia acclimation pathway and its regulation by a broader range of conditions suggest an ability to integrate different signals with hypoxia acclimation, which could be beneficial for multi-stress tolerance. To disentangle the effects of PGB1 on NO homeostasis from its NO-independent contribution to hypoxia tolerance networks, a comprehensive analysis of PGB1-mediated regulation of NO signaling is needed. For example, the PGB1-dependent changes in protein *S*-nitrosylation profiles could be investigated using both loss-of-function mutants and overexpression lines in stress-specific contexts.

An important unresolved question is also the extent to which the biological functions of PGB1 are mediated through its distinct downstream pathways. This could be addressed by combining genetic resources and approaches. For instance, the contribution of PGB1 to ERF-VII-independent or MRR-independent pathways could be assessed by analyzing PGB1 function or overexpression in, for example, an *erfvii* or *anac* mutant background. Likewise, examining the effects of ethylene-induced *PGB1* expression in these genetic contexts would help to disentangle the ERF-VII-dependent pre-acclimation mechanisms from other PGB1-mediated processes.

In conclusion, PGB1 is a crucial player in hypoxia signaling and acclimation, and continued research on its functional properties, regulatory mechanisms, and physiological roles promises to yield insights of relevance for improving plant stress resilience.

## Abbreviations

ABA: abscisic acid
ERF-VII: ETHYLENE RESPONSE FACTOR VII
LR: lateral root
MDHAR: monodehydroascorbate reductase
MED25: MEDIATOR SUBUNIT25
MRR: mitochondrial retrograde regulation
NAC: NO APICAL MERISTEM/ARABIDOPSIS TRANSCRIPTION ACTIVATION FACTOR/CUP-SHAPED COTYLEDON
NO: nitric oxide
PAT: polar auxin transport
PCO: PLANT CYSTEINE OXIDASE
PGB: phytoglobin
PGB1: class I phytoglobin
PGB2: class II phytoglobin
PGB3: class III phytoglobin
PIN: PIN-FORMED
PRT6: PROTEOLYSIS6
QC: quiescent center
ROS: reactive oxygen species
symPGBs: symbiotic phytoglobins
